# CT findings of patients infected with SARS-CoV-2

**DOI:** 10.1186/s12880-020-00471-6

**Published:** 2020-06-23

**Authors:** Xiaoyang Wang, Chenbin Liu, Liang Hong, Cuiyun Yuan, Jiguang Ding, Qing Jia, Gangqiang Sun, Wenxian Peng, Qingfeng Sun

**Affiliations:** 1 Department of Radiology, Ruian People’s Hospital, Wenzhou, 325200 China; 2 Department of Radiation Oncology, National Cancer Center/National Clinical Research Center for Cancer/Cancer Hospital & Shenzhen Hospital, Chinese Academy of Medical Sciences and Peking Union Medical College, Shenzhen, 518116 China; 3 Department of Infectious Disease, Ruian People’s Hospital, Wenzhou, 325200 China; 4 Department of Biology, Gordon College, Wenham, MA 01984 USA; 5 College of Medical Imaging, Shanghai University of Medicine and Health Sciences, Shanghai, 201318 China

**Keywords:** CT imaging, SARS-CoV-2, COVID-19

## Abstract

**Background:**

We aimed to describe the chest CT findings in sixty-seven patients infected by Severe acute respiratory syndrome coronavirus 2 (SARS-CoV-2).

**Methods:**

We retrospectively reviewed 67 patients hospitalized in Ruian People’s Hospital. All the patients received the positive diagnosis of SARS-CoV-2 infection. The CT and clinical data were collected between January 23rd, 2020 and February 10th, 2020. The CT images were analyzed by the senior radiologists.

**Results:**

There are 54 patients with positive CT findings and 13 patients with negative CT findings. The typical CT findings in hospitalized patients with SARS-CoV-2 infection were ground glass opacities (42/54), lesions located in the peripheral area (50/54), multiple lesions (46/54), and lesions located in the lower lobes (42/54). There were less typical CT findings, including air bronchogram (18/54), pleural thickening or pleural effusion (14/54), consolidation (12/54), lesions in the upper lobes (12/54), interlobular septal thickening (11/54), reversed halo sign (9/54), single lesion (8/54), air cavities (4/54), bronchial wall thickening (3/54), and intrathoracic lymph node enlargement (2/54).

**Conclusions:**

CT features can play an important role in the early diagnosis and follow-up of COVID-19 patients.

## Background

Since December 8th, 2019, several pneumonia cases with unknown causes were reported in Wuhan, China. The disease has rapidly spread around the whole country and even the world [1–3]. In February 2020, an increased number of patients infected with this severe acute respiratory syndrome coronavirus 2 (SARS-CoV-2) were reported around the world. Most clinical studies focused on clinical and epidemiological features of the patients infected with SARS-CoV-2 [4, 5]. The most common onset symptoms were fever, cough, and myalgia or fatigue; less common symptoms were sputum production, headache, hemoptysis, and diarrhea [4]. However, there are only a few reports about computed tomography (CT) findings of the patients infected with SARS-CoV-2.

Coronaviruses may produce specific features in chest CT images, such as severe acute respiratory syndrome (SARS) and middle east respiratory syndrome (MERS) [6–9]. The typical CT findings in patients infected by SARS were ground glass opacities [9]. The SARS lesions can progress rapidly to focal, multifocal, or diffuse consolidation [9]. The most common CT findings in patients infected with MERS-CoV was subpleural and basilar airspace changes [8]. In our hospital, a chest CT scan was widely used in the diagnosis and follow-up evaluation of patients infected with SARS-CoV-2. The purpose of this study was to describe the chest CT findings of laboratory-confirmed SARS-CoV-2 cases.

## Methods

### Patients

The approval for this study was obtained from the Institutional Review Boards of Ruian People’s hospital. The informed consent was waived. Sixty-seven patients infected by novel coronavirus were retrospectively collected from Ruian People’s Hospital since January 21st, 2019. We collected demographic, clinical, laboratory, treatment, and prognostic data for all patients and followed up to February 15th, 2020. The diagnosis of coronavirus disease 2019 (COVID-19) was established according to China’s National Health Commission criteria. The oropharyngeal swabs and deep cough sputum were tested by the 2019 new coronavirus (ORF lab/E/N gene) nucleic acid detection kit (Shanghai BioGerm Medical Biotechnology Co., Ltd).

### Imaging techniques

The chest CT scan was performed using one of the following multi-slice spiral CT scanners: Siemens SOMATOM Perspective CT Scanner (Siemens Medical Solution, Forchheim, Germany), 32-slice configurations; uCT 528 (United Imaging Healthcare, Shanghai, China), 40-slice configurations. Scanning parameters were shown as follows: tube voltage, 120 kV; adaptive tube current, 100 ~ 350 mA; slice thickness, 1.5 mm for Siemens CT, 1.0 mm for uCT.

### Image analysis

CT images were analyzed on a radiology PACS workstation (Greenlander version 6.0, Mindray Healthcare, Shenzhen, China). All chest CT examinations were reviewed independently by two senior radiologists. A third senior radiologist was consulted when there were different opinions between the two radiologists. The CT imaging features were assessed, including ground glass opacities (GGO), consolidation, air bronchogram, reversed halo sign, interlobular septal thickening, and subpleural linear shadow. The transverse distributions of abnormalities were categorized as peripheral and central. The lesions in the outer third and inner two-thirds of the lungs were defined as peripheral and central lesions, respectively. The distribution of lesions in the middle-upper and lower lobes were also recorded. In addition, we also observed the presence of tree-in-bud pattern, air cavity, bronchial wall thickening, intrathoracic lymph node enlargement, and pleural effusion.

## Result

As shown in Table 1, there were a total of 67 COVID-19 patients (31 males and 36 female patients) with an age range of 5–72 years old (median age, 44 years old). Most of the patients infected with SARS-CoV-2 have lesions in the chest CT images (54/67). Common symptoms at the onset of illness were fever (58/67), cough (52/67), and sputum production (35/67). Less common symptoms were chest tightness (12/67), Sore throat (8/67), diarrhea (5/67), dizziness (3/67), shortness of breath (4/67), nausea and vomiting (2/67), myalgia or fatigue (3/67), and headache (2/67). For the job type, 59 patients were self-employed, four patients were farmers, and four patients were company employees. For the type of infection source, 34 patients lived in Wuhan, three patients lived in Hubei province, 27 patients had a history of contact with COVID-19 patients, two patients had a history of contact with suspected patients, and only one patient had no history of contact with COVID-19 patients or suspected patients. For the smoking status, two patients were former smokers, three patients were current smokers, and the remaining sixty-two patients were non-smokers. There are sixteen patients with one or more of the following comorbidities: hypertension (11/16), diabetes (7/16), cancer (1/16), respiratory disease (1/16), cardiovascular disease (1/16), and connective tissue disease (1/16). After the treatment, 44 patients were cured, and the remaining 23 patients were still hospitalized.

**Table 1:** Demographic and clinical characteristics of 67 patients with SARS-CoV-2

	Number
Gender (male)	31 (46.3%)
Positive CT findings	56 (83.6%)
Age	44 (5 ~ 72)
Job type	4 (6.0%) farmers, 59 (88.0%) self-employed, 4 (6.0%) company employees
Infection source type	34 (50.8%) lived in Wuhan, 3 (4.4%) lived in Hubei Province, 27 (40.3%) got contact with patients, 2 (3.0%) got contact with suspected patient, 1 (1.5%) unknown
Smoking status	62 (92.5%) are non-smokers, 2 (3.0%) are former smokers, 3 (4.5%) are current smokers
Severe patients	3 (4.5%)
Fever	58 (86.6%)
Cough	52 (77.6%)
Sputum production	35 (52.2%)
Chest tightness	12 (17.9%)
Sore throat	8 (11.9%)
Diarrhea	5 (7.5%)
Dizziness	3 (4.5%)
Shortness of breath	4 (6.0%)
Nausea and vomiting	2 (3.0%)
Myalgia or fatigue	3 (4.5%)
Headache	2 (3.0%)
Current status (cured/hospitalized)	44 (65.7%) cured, 23 (34.3%) hospitalized

**Fig. 1:**
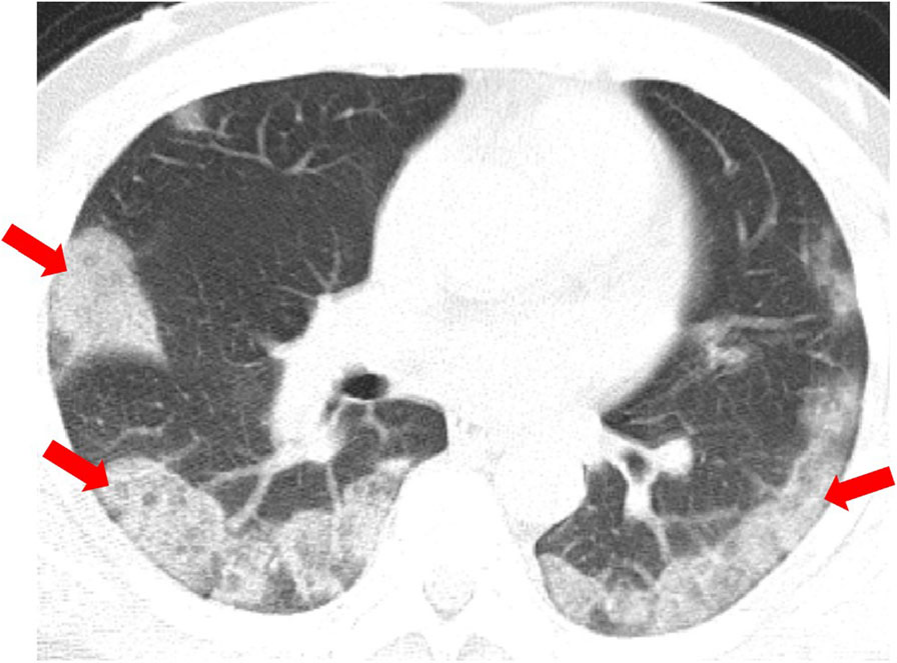
A male patient infected with SARS-CoV-2 who presented severe difficulty in breathing. Transverse CT performed on six days after onset of symptoms shows peripheral ground glass opacities (red arrow) under the pleura of the middle and lower lobes in both lungs

**Fig. 2:**
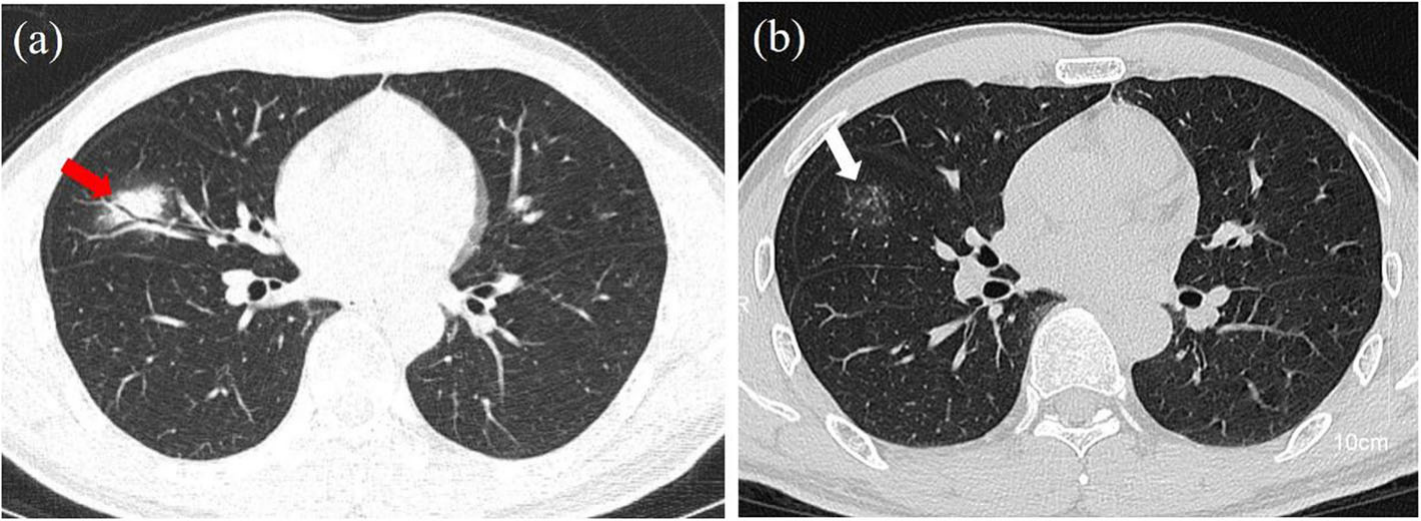
A male patient infected with SARS-CoV-2. **a** The first CT was performed three days after the onset of symptoms. The chest CT image shows single consolidation in the right middle lobe and air bronchogram (red arrows). **b** The second CT was performed 13 days after the treatment. The chest CT image shows a tree-in-bud pattern (white arrows)

**Fig. 3:**
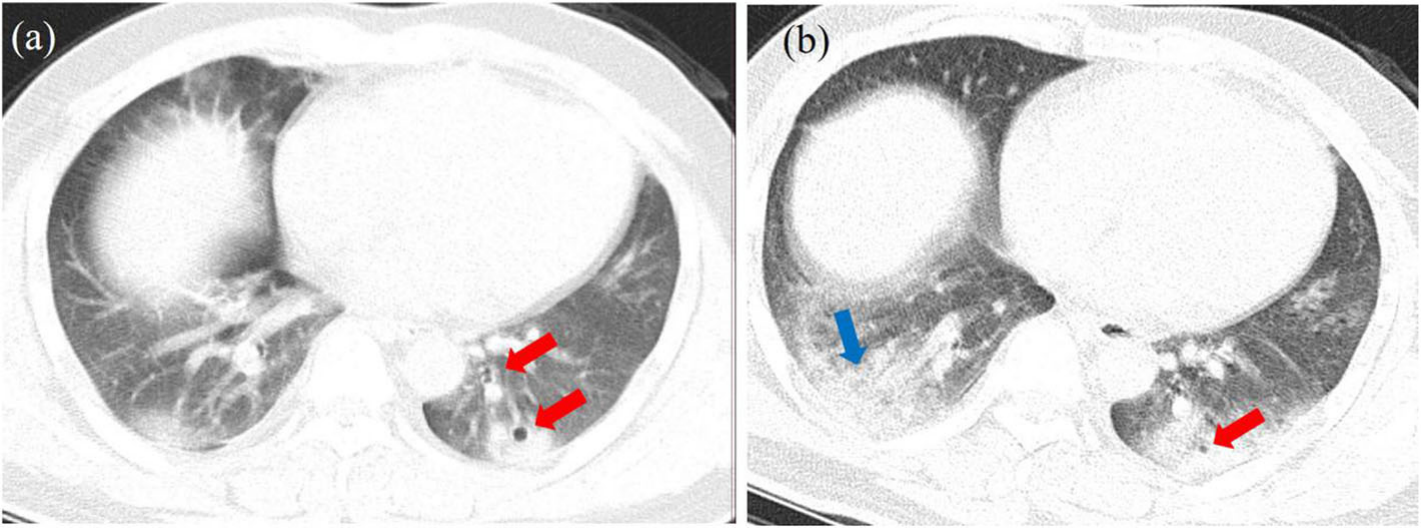
A male patient infected with SARS-CoV-2. **a** The first CT was performed three days after the onset of symptoms. The chest CT image shows ground glass opacities in the lower lobe and air cavities (red arrows). **b** The second CT was performed three days after the treatment. The chest CT image shows ground glass opacities occupying the left lower lobe (blue arrow)

**Fig. 4:**
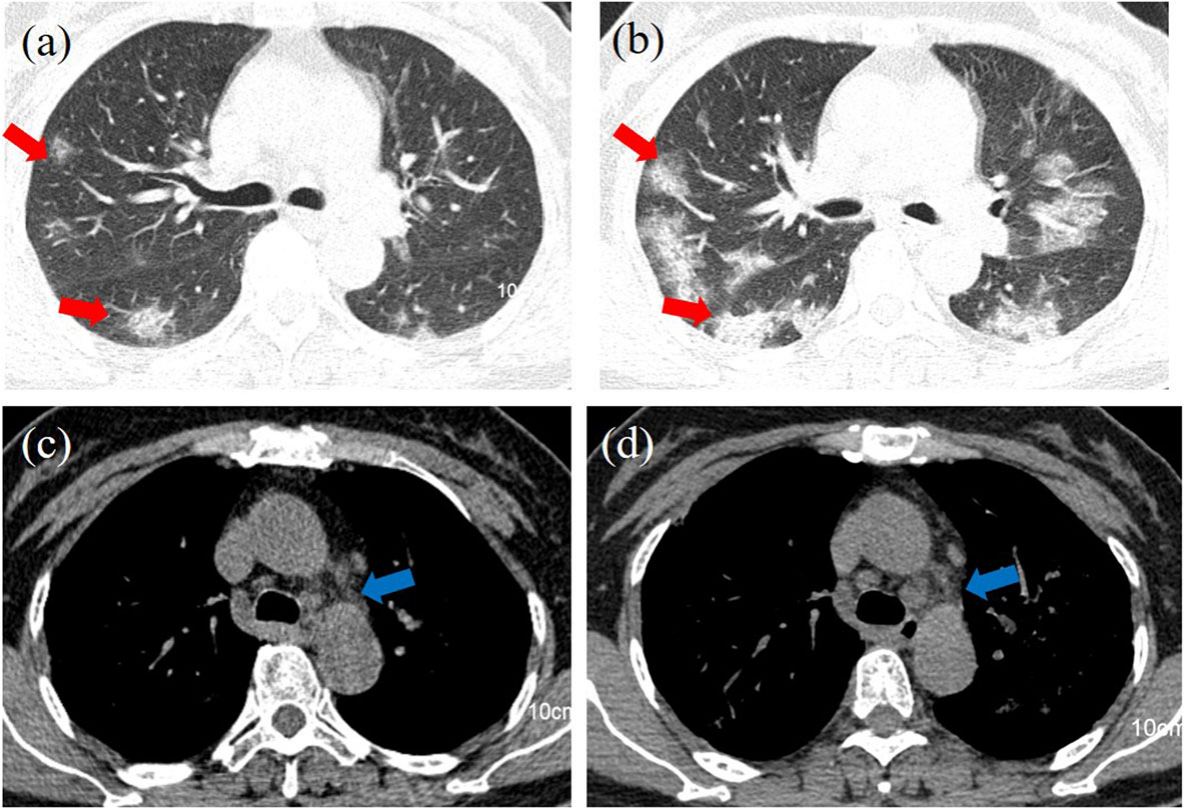
A female patient infected with SARS-CoV-2. The first CT scans (**a**) and (**c**) were performed four days after onset of symptoms. The second CT scans (**b**) and (**d**) were performed ten days after the treatment. Chest CT images showed the enlargement of ground glass opacity (red arrows) and intrathoracic lymph node (blue arrows) after 10-day treatment

**Fig. 5:**
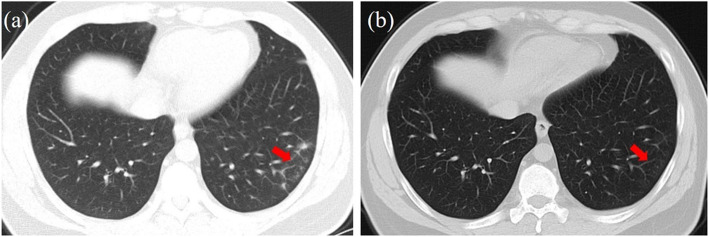
A male patient infected with SARS-CoV-2. **a** The first CT was performed three days after the onset of symptoms. The chest CT image shows a tree-in-bud pattern (red arrow). **b** The second CT was performed two weeks after the treatment. The chest CT image shows the tree-in-bud pattern was fully absorbed after the treatment

All the patients underwent CT 1–11 days after admission (median, 4 days). There were 54 of 67 patients with lesions, and the remaining 13 patients were normal in chest CT images. As shown in Table 2, fifty of the fifty-four patients had lesions in the peripheral regions (Fig. 1), and four patients had lesions in the central areas. Twelve patients had abnormalities in the middle-upper lobe, and the remaining forty-two patients had abnormalities in the middle-lower lobe. There were eight patients with a single lesion (Fig. 2(a)) and 46 patients with multiple lesions (Fig. 1). Forty-two of the fifty-four patients had ground-glass opacities (Figs. 1, 2, 3, 4), and twelve patients had isolated consolidation (Fig. 2(a)). Interlobular septal thickening was identified in eleven patients. Reversed halo sign was noted in nine patients. There were eighteen patients with air bronchogram. Three patients had bronchial wall thickening. Tree-in-bud pattern was identified in one patient (Fig. 5(a)). Air cavities were present in four patients (Fig. 3). Pleural thickening or pleural effusion was noted in fourteen patients. Only two patients had intrathoracic lymph node enlargement (Fig. 4 (d)).

**Table 2:** CT features in sixty-seven patients with SARS-CoV-2

	Number
Peripheral/central lesions	50 (92.6%) peripheral; 4 (7.4%) central
Lesions in middle upper/lower lobes	12 (22.2%) upper lobe; 42 (77.8%) lower lobe
Single/multiple lesions	8 (14.8%) single; 46 (85.2%) multiple
Ground-glass opacities	42 (77.8%)
Consolidation	12 (22.2%)
Interlobular septal thickening	11 (20.4%)
Reversed halo sign	9 (16.7%)
Air bronchogram	18 (33.3%)
Bronchial wall thickening	3 (5.6%)
Tree-in-bud pattern	1 (1.9%)
Air cavity	4 (7.4%)
Pleural thickening or pleural effusion	14 (25.9%)
Intrathoracic lymph node enlargement	2 (3.7%)

## Discussion

This study shows that multiple peripheral lesions on CT are common in patients hospitalized with SARS-CoV-2 infection. Ground-glass opacities were more extensive than consolidation in most patients. The average time interval between the onset of symptoms and the CT examination was 4.6 days. It suggested that the CT findings in our study were typical for COVID-19 patients in the early stage. Given patients with a history of exposure and the imaging features mentioned above, SARS-CoV-2 infection would be highly suspected. Further tests will be necessary. As the exposure history became complicated and unclear over time, the chest CT scans would play an essential role in the diagnose of COVID-19. However, there were still 13 COVID-19 patients with negative chest CT findings. As far as we know, both CT and nucleic acid detection are indispensable in the diagnose.

In our study, four patients had small cavities in the chest CT (Fig. 3), and their clinical symptoms were relatively acute. The short-term progress of chest CT imaging was somewhat evident (Fig. 3). Our study reported that a tree-bud pattern was identified in one case before the treatment and fully recovered after the treatment. Thus, the tree-bud pattern was not considered to be a typical CT feature caused bySARS-CoV-2 due to the short duration of the COVID-19 presence. Distinguished from previous reports of viral pneumonia, air cavity, bronchial wall thickening, and pleural effusion were found in the patients infected with SARS-CoV-2 (Table 2). In fifty-four positive CT finding cases, there are 5 cases with a single lesion, and the maximum of major axis length is 23 mm. To our knowledge, a single lesion located in the middle of the lung with the major axis length over 30 mm is possibly not a lesion of COVID-19.

After the treatment, thirty-nine of 67 patients infected with SARS-CoV-2 were cured and discharged. Repeated nucleic acid tests were performed to show SARS-CoV-2 clearance before hospital discharge. The chest CT scans before hospital discharge were also partially or fully recovered compared to the first ones after hospitalization. The partial recovery in lung lesions could be caused by the clinical lag of lung imaging. We also noticed that there were 13 cases with the progress of CT findings during the treatment, and ten of the 13 cases recovered in CT features after 10-day treatment.

There are two limitations in our study. First, the number of our patient cohort was small. Only a small amount of severe patients were presented. Recently, a large number of patients were confirmed to be infected with SARS-CoV-2. We will collect the clinical and CT features of a large patient population in future studies. Second, we did not perform a sequential CT study due to the urgency of sharing our CT findings of this new disease. In the next step, we will further evaluate the longitudinal change of CT features during the treatment. A predictive model of the clinical outcome will be developed based on CT imaging.

## Conclusion

In summary, CT features of patients with SARS-CoV-2 were predominantly subpleural multiple ground glass opacities in two lungs, some with consolidations. There are also less common CT features, such as air bronchogram and reversed halo sign. It is rare to observe some features, such as bronchial wall thickening, tree-in-bud pattern, and pleural effusion. The CT features can play an important role in the early diagnosis and follow-up of COVID-19 patients.

## Supplementary information

**Additional file 1.**

## Data Availability

The data analysed during this study are included in this published article and its supplementary information files. The age and gender information was removed to protect the patient privacy. The raw data may be made available upon reasonable request from the corresponding author.

## Abbreviations

SARS-CoV-2: Severe acute respiratory syndrome coronavirus 2
CT: Computed tomography
COVID-19: Coronavirus disease 2019
SARS: Severe acute respiratory syndrome
MERS: Middle East respiratory syndrome
PACS: Picture archiving and communication system
GGO: Ground glass opacities
